# Applying Abstract Text Mining as a Complement to PRISMA in Reviewing the Scope of Healthcare’s Circular Economy: Comment on "A Review of the Applicability of Current Green Practices in Healthcare Facilities"

**DOI:** 10.34172/ijhpm.9410

**Published:** 2026-02-02

**Authors:** Amin Esmaeili

**Affiliations:** Industrial and Systems Engineering Department, Kennesaw State University, Marietta, GA, USA.

**Keywords:** Global Warming, Climate Change, Hospital, Medical, Clinics, Environmental Impact

## Abstract

Efforts to reduce the healthcare sector’s carbon footprint and greenhouse gas (GHG) emissions have brought increased attention to the adoption of the circular economy (CE) in recent years. These efforts aim to lower carbon-intensive products while improving efficiency, waste reduction, and healthcare resilience. Soares et al conducted a scoping review examining CE applicability in healthcare and identified strategies to enhance its implementation. In this commentary paper, a novel abstract text mining (ATM) approach is introduced as a complement to the standard Preferred Reporting Items for Systematic Reviews and Meta-Analyses (PRISMA) methodology. Using this approach, the search terms employed by Soares et al were expanded, article abstracts were extracted, and scope areas were mapped with the assistance of a well-established machine learning technique—latent Dirichlet allocation (LDA) topic modeling. Comparison of the ATM results with those reported by Soares et al revealed three additional scope areas: alternative treatment pathways, pharmaceutical footprint reduction, and the utilization of emerging technologies.

## Introduction

 The healthcare sector contributes 4.4% of global greenhouse gas (GHG) emissions^1^ and, therefore, the urgent need to cut its carbon footprint has gained the attention of healthcare researchers over the past two decades. The concept of the circular economy (CE) offers a framework to address this challenge. While it is a relatively recent framework, it is rooted in earlier ideas such as industrial ecology, cradle-to-cradle design, and environmental sustainability.^2^ In the context of healthcare, adopting CE principles could reduce reliance on carbon-intensive production of medical devices, pharmaceuticals, and single-use products, as well as support broader sustainability goals such as promoting waste reduction, resource and energy efficiency, and resilience. In 2023, Soares et al conducted a scoping review to examine the applicability of CE in hospitals^3^ and to identify strategies for improving its implementation in healthcare. In this commentary paper, the aim is to expand Soares and colleagues’ findings by identifying additional scope areas that could be used for future environmental sustainability and CE research topics by scholars.

## Summary of the Findings Reported by Soares et al

 Soares et al organized their paper very well. First, a background and overview of the implementation of CE in the European Union region were summarized, and then they examined the applicability of CE in the overall healthcare sector and within hospitals in two separate sections. In the overview of the healthcare sector, they referenced many highly cited articles from Europe, the UK, the USA, Canada, China, Japan, and Austria, and reviewed those studies to create their scoping review article. In the application of CE within hospitals, Soares et al suggested opportunities related to hospital building design, green team establishment, waste management, energy, water, food consumption reduction, transportation, telemedicine, procurement, and behavior.

## Broadening Keyword Selection

 The search strategy used by Soares et al in their scoping review was based on the Preferred Reporting Items for Systematic Reviews and Meta-Analyses (PRISMA) guidelines.^3^ PRISMA is one of the most widely used methods for conducting systematic literature reviews, and their use of three major databases—MEDLINE, Scopus, and Web of Science—adds confidence that the initial pool of articles included relevant literature for the chosen keywords: (“circular economy” OR “carbon footprint”) AND (“hospital” OR “healthcare”). However, their search strategy could be improved by including additional synonyms such as “greenhouse gas (GHG),” “clinics,” “medical facilities,” or other related terms. This is particularly relevant because the term “GHG” appears more than 40 times in their article, often used interchangeably with “carbon footprint.” To demonstrate the potential impact of widening the keyword selection, a search was conducted in the Scopus database using an expanded set of terms, which identified 377 additional articles published between 1992 and 2022. The list of these additional articles can be generated in the Scopus database by using the following query.

 ((TITLE-ABS-KEY(circular economy) OR TITLE-ABS-KEY(carbon footprint) OR TITLE-ABS-KEY (greenhouse gas)) AND (TITLE-ABS-KEY(hospital) OR TITLE-ABS-KEY(healthcare) OR TITLE-ABS-KEY(clinic) OR TITLE-ABS-KEY(medical facility)) AND PUBYEAR > 1991 AND PUBYEAR < 2023) AND NOT ((TITLE-ABS-KEY(circular economy) OR TITLE-ABS-KEY(carbon footprint)) AND (TITLE-ABS-KEY(hospital) OR TITLE-ABS-KEY(healthcare)) AND PUBYEAR > 1991 AND PUBYEAR < 2023) AND (LIMIT-TO (DOCTYPE, “ar”) OR LIMIT-TO (DOCTYPE, “re”) OR LIMIT-TO (DOCTYPE, “cp”)) AND (LIMIT-TO (LANGUAGE, “English”))

 Reviewing the abstracts of the highly-cited papers among these 377 additional articles revealed studies on outpatient services that are relevant to green healthcare but were excluded from the study by Soares et al, as their authors did not use the terms hospital or healthcare in their titles, abstracts, or keywords. Examples include studies by Edison et al^4-6^ on urology, Duane et al^7,8^ on dental practice, Sehgal et al^9^ on hemodialysis, Mojdehbakhsh et al^10^ on gynecologic oncology clinics, Goel et al^11^ on cataract surgeries, and Penaskovic et al^12^ on telepsychiatry.

 These papers also indicate the need for an expansion of Soares and colleagues’ scoping review of CE applications in healthcare. As noted by Devlin-Hegedus et al^13^ and Unger et al,^14^ a comprehensive framework and decision-support system are needed to inform healthcare providers about environmentally friendly treatment alternatives that deliver equivalent patient-care outcomes. Following this approach, many scholars have focused on specific clinical practices and quantified GHG emission reductions associated with alternative treatments in nephrology,^15-17^ eye care services,^18^ gastroenterology and hepatology,^19^ pathology,^20^ anesthesia,^21-24^ hand surgery,^25^ and initiatives in operating rooms.^26-28^ However, these practice-focused studies did not explicitly use the terms carbon footprint or CE and, therefore, were not included in Soares and colleagues’ pool of reviewed articles. Additionally, the use of the term GHG enabled the identification of several studies related to medical waste management^29-33^ and healthcare-related transportation,^34-36^ which can strengthen two of the topics discussed by Soares et al study.

 According to the methodological reference of the Soares et al paper,^37^ the primary goal of a scoping review is to map the existing body of literature on a topic by clarifying key concepts and identifying the main sources and types of evidence available. The inclusion of these additional 377 papers into the article pool would require screening and subsequent full-text assessment of selected studies in order to update the topic mapping. As this time-consuming process falls outside the scope of this present commentary, this paper instead introduces a novel approach that leverages a well-established machine learning technique to cluster the literature. This approach is named abstract text mining (ATM), and other instances of its use can be found in future works by the corresponding author.

## Expansion of Scope Areas

 To conduct a gap analysis and assess the adequacy of the topics mapped by Soares et al in relation to CE and green healthcare improvement areas, a pool of 2560 articles published between January 1992 and December 2025 was compiled. These articles were identified using the first part of the aforementioned Scopus search query, which included the all the terms (circular economy OR carbon footprint OR greenhouse gas) AND (hospital OR healthcare OR clinic OR medical facility). The temporal boundary of the query was also extended to 2025 to capture recently published studies, and Figure shows that the increasing rate of relevant publications has continued after 2022.

**Figure:**
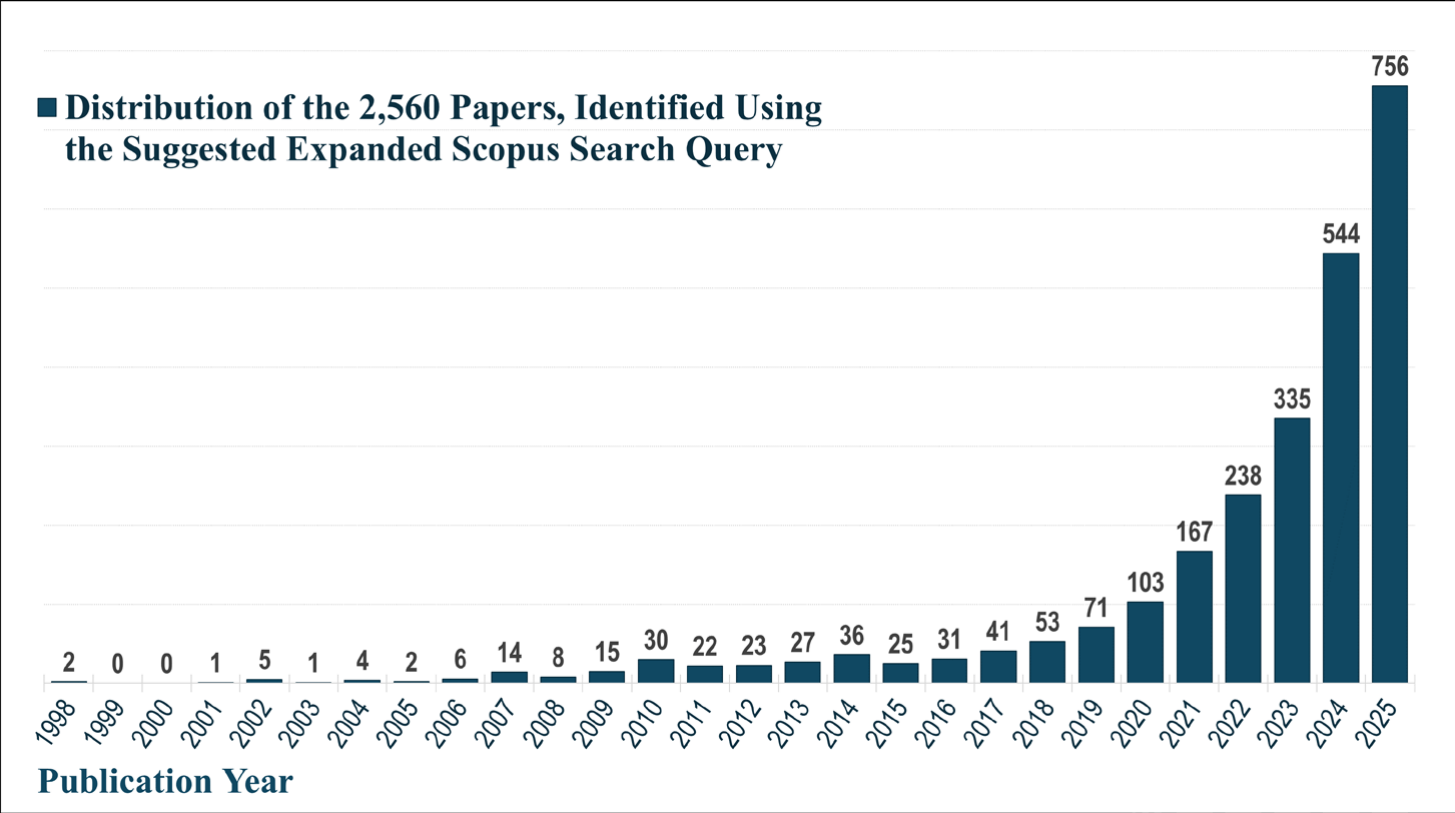
Distribution of the 2560 Articles Containing Expanded Search Terms in Their Title, Abstract, or Keywords.

 The abstracts of these papers were then used as the input corpus for text mining method of latent Dirichlet allocation (LDA) topic modeling, which is an unsupervised machine learning approach based on probabilistic clustering.^38^ The abstracts of the 2560 articles were preprocessed using the R tm text mining package to construct the document–term matrix. During document–term matrix construction, words were reduced to their root forms using the stemming algorithm implemented in the R SnowballC package, ensuring that different grammatical variants of the same word were treated as a single term in the text analysis. The LDA algorithm, implemented with 3000 iterations of Gibbs sampling using the R topicmodels package, was then applied to cluster the 2560 papers into 40 topics. Finally, the R wordcloud package was used to generate visual representations of these 40 topics, which are presented in Table. Topic labels were assigned based on the dominant stemmed terms within each cluster, and topic rankings were determined according to their similarity to the scope areas mapped by Soares et al. Table also reports the percentage of the 2560 papers assigned to each topic.

**Table:** Word Clouds, Assigned Titles, and Percentage of the 2560 Articles Across Topics

**Topic Title**	**Word Cloud**	**% Of Total**	**Topic Title**	**Word Cloud**	**% Of Total**
1. Hospital Waste Management and Recycling	**Figure:** 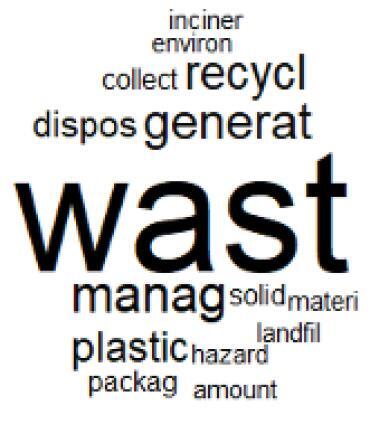	4.5%	21. Anesthesia Gases and Clinical Emissions	**Figure:** 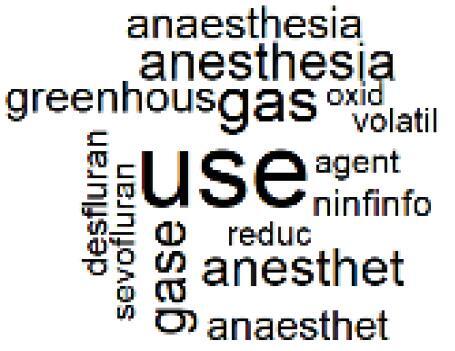	3.7%
2. Biomedical Material and Device Manufacturing	**Figure:** 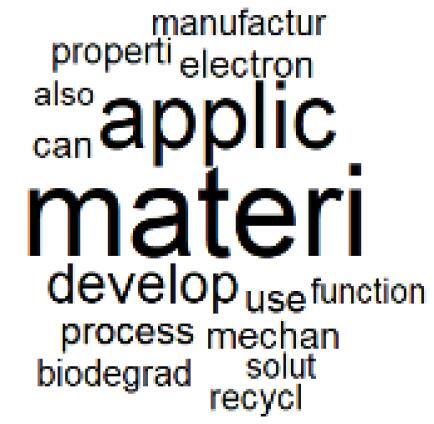	3.4%	22. Chemical Processes of Pharmaceutical	**Figure:** 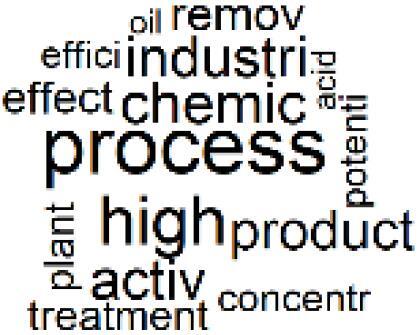	4.0%
3. Reusable vs. Single-Use Medical Devices	**Figure:** 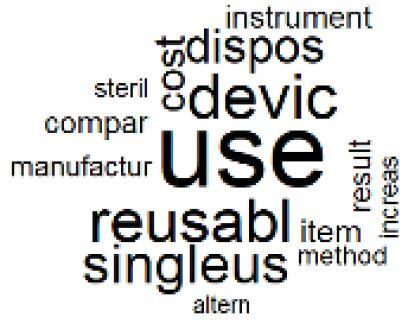	2.5%	23. Smart Healthcare Technologies and Efficiency	**Figure:** 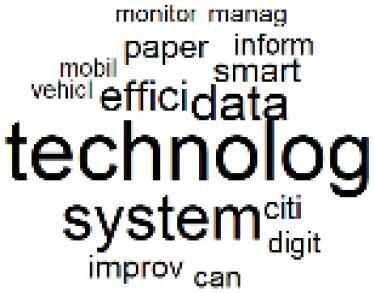	2.7%
4. Supply Chains and CE	**Figure:** 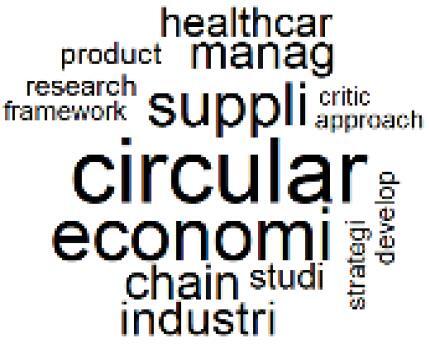	3.2%	24. Analytical Models and Decision-Support Tools	**Figure:** 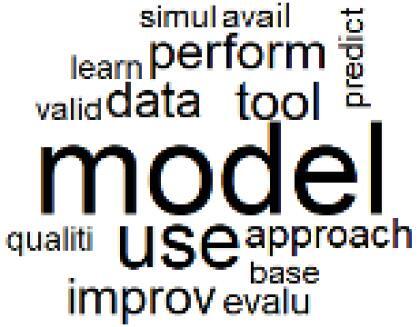	1.8%
5. GHG Emissions and Transportation	**Figure:** 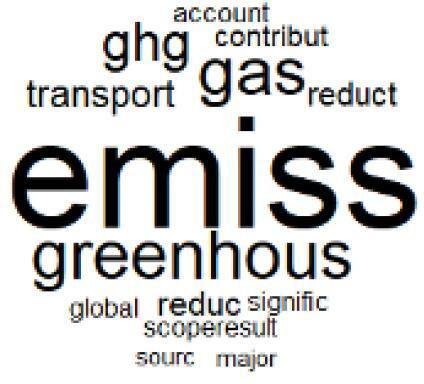	2.0%	25. COVID-19 Environmental Impact	**Figure:** 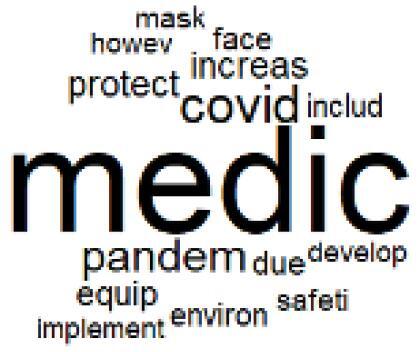	1.4%
6. Telemedicine and Patient Travel Reduction	**Figure:** 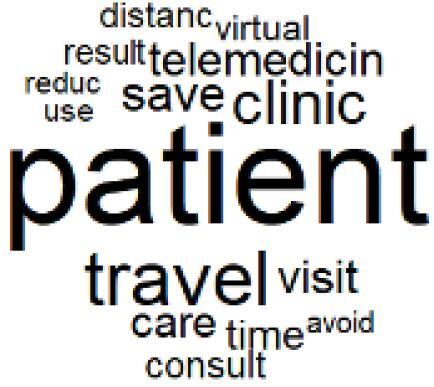	4.1%	26. Cost Modeling Optimization	**Figure:** 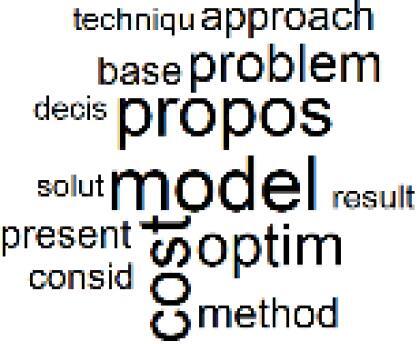	2.4%
7. Energy Consumption Reduction in Facilities	**Figure:** 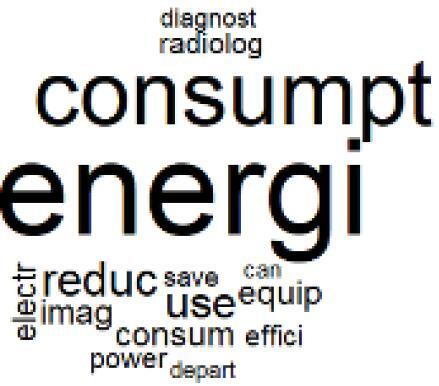	2.0%	27. Cost Reduction of Clinical Interventions	**Figure:** 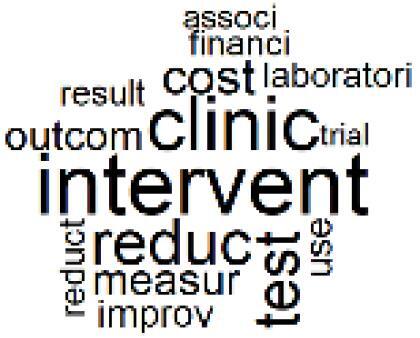	1.6%
8. Renewable and Energy Systems	**Figure:** 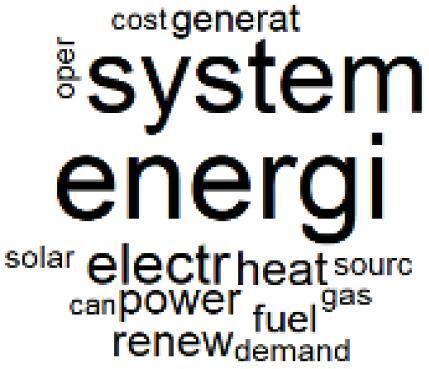	4.5%	28. Public Health and Healthcare Systems	**Figure:** 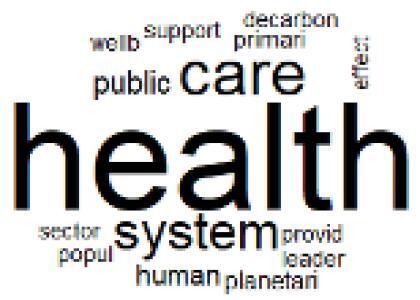	2.1%
9. Sustainable Buildings and Facilities	**Figure:** 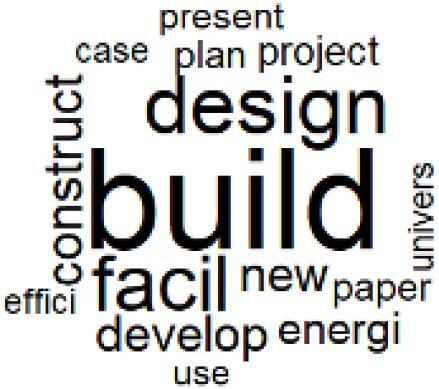	2.5%	29. Climate Change and Global Health	**Figure:** 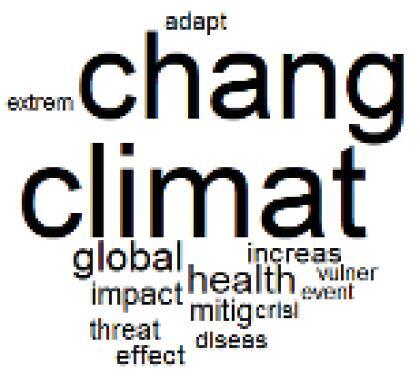	4.0%
10. Food Systems Environmental Impact	**Figure:** 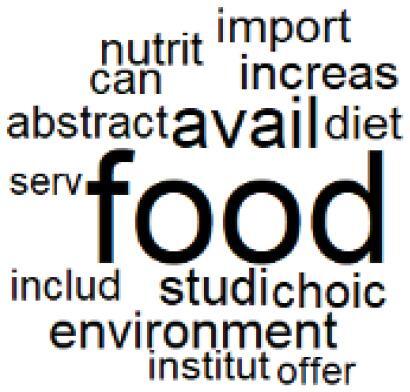	3.6%	30. Ecological Role of the Healthcare Sector	**Figure:** 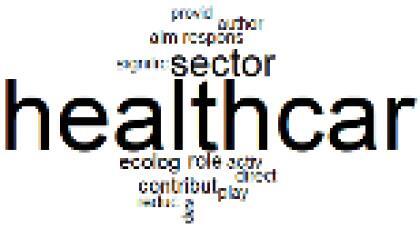	0.9%
11. Water and Resource Use in Healthcare	**Figure:** 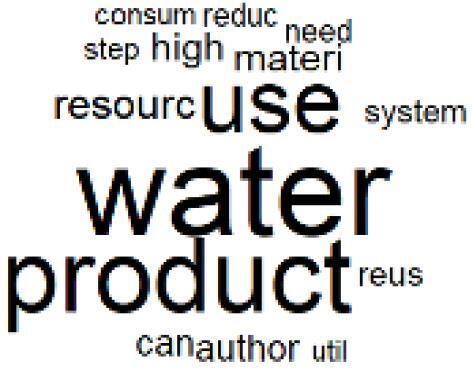	1.2%	31. Air Pollution and Health Impact	**Figure:** 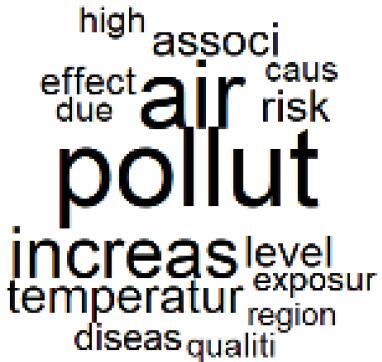	2.7%
12. Education, Awareness, and Sustainability Barriers	**Figure:** 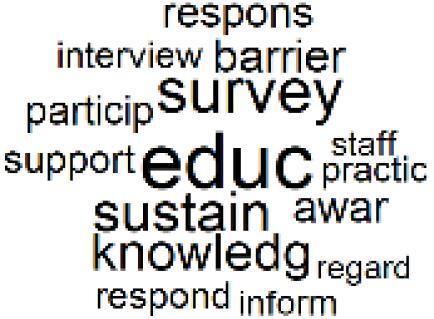	3.9%	32. Worldwide Healthcare Environmental Solutions	**Figure:** 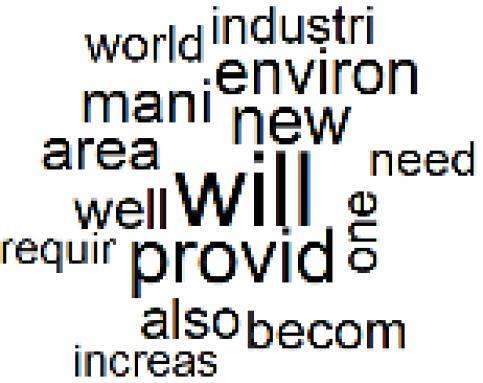	1.5%
13. Green Healthcare Practices	**Figure:** 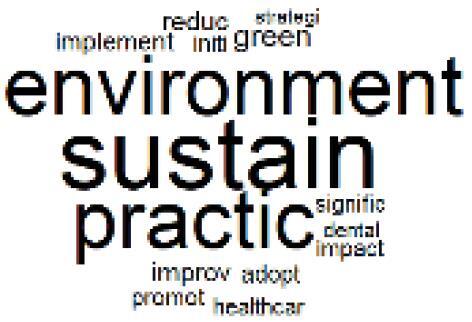	2.3%	33. Ethics, Society, and Scientific Publishing	**Figure:** 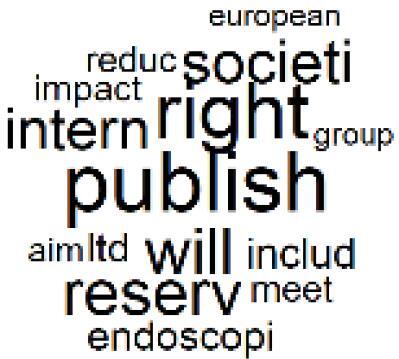	1.0%
14. Patient Care and Treatment Pathways	**Figure:** 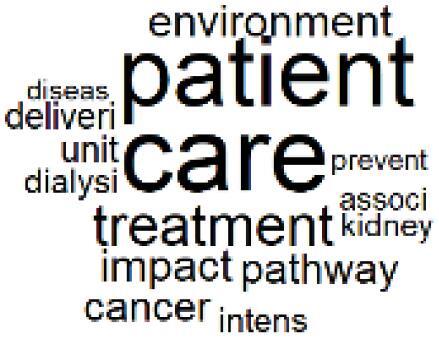	3.0%	34. NHS Initiatives	**Figure:** 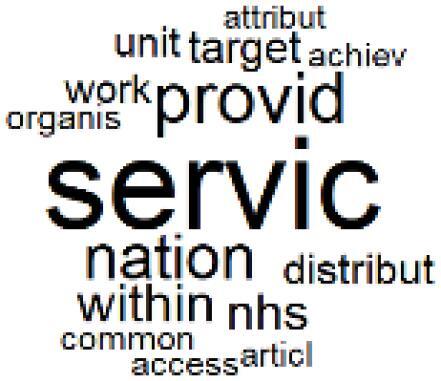	1.6%
15. Accounting GHG Emission Estimation	**Figure:** 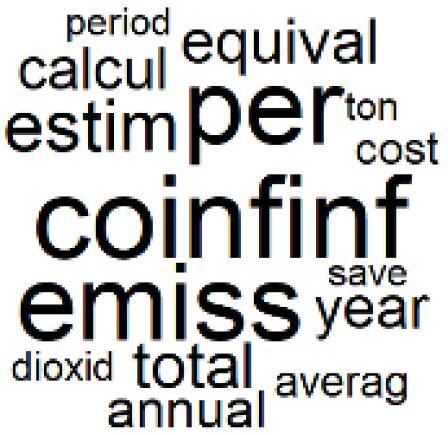	2.3%	35. Literature Reviews and Evidence Mapping	**Figure:** 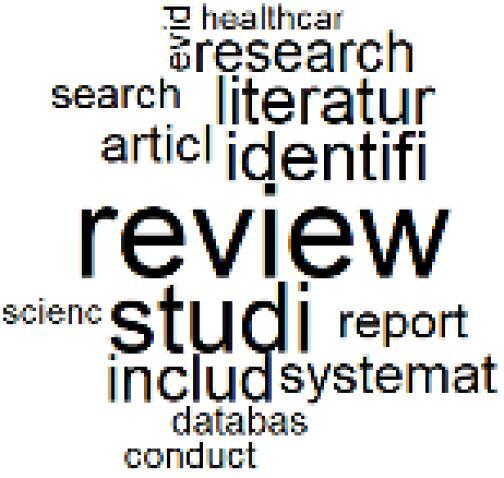	3.8%
16. Life Cycle Environmental Assessment	**Figure:** 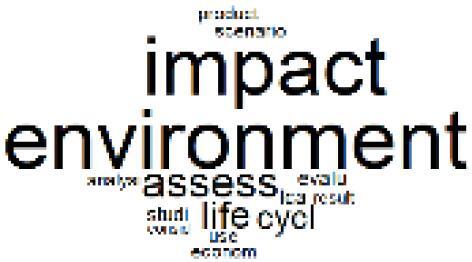	2.5%	36. General Environmental Research Trends	**Figure:** 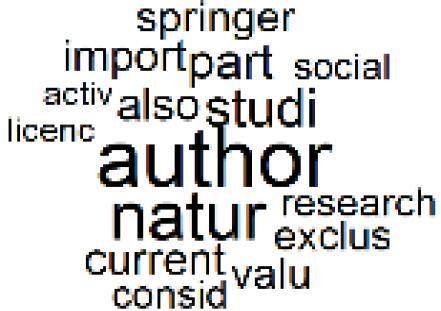	0.9%
17. Carbon Footprint Analysis	**Figure:** 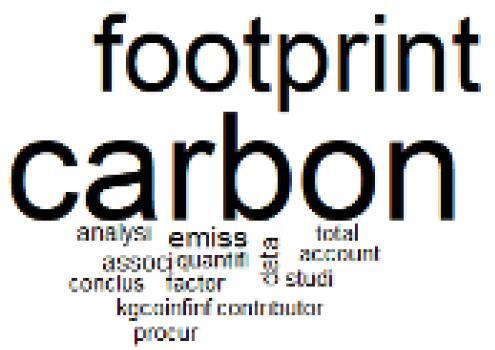	1.6%	37. Healthcare Sustainability Integration Research	**Figure:** 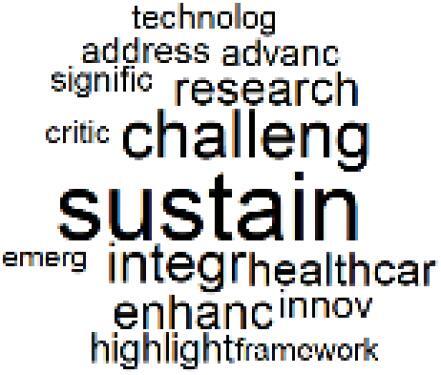	3.6%
18. Comparative Impact Studies	**Figure:** 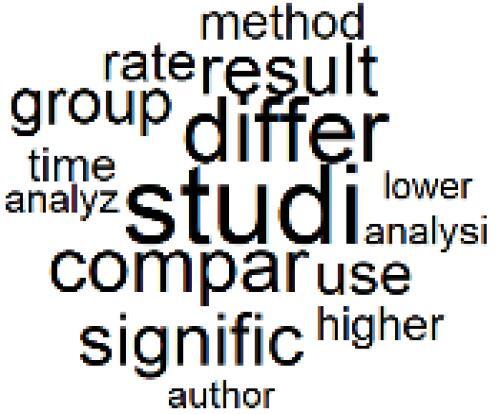	0.6%	38. Healthcare Policy and Economic Development	**Figure:** 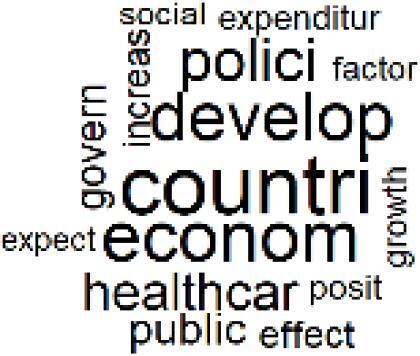	2.3%
19. Surgical Operations and Operating Rooms	**Figure:** 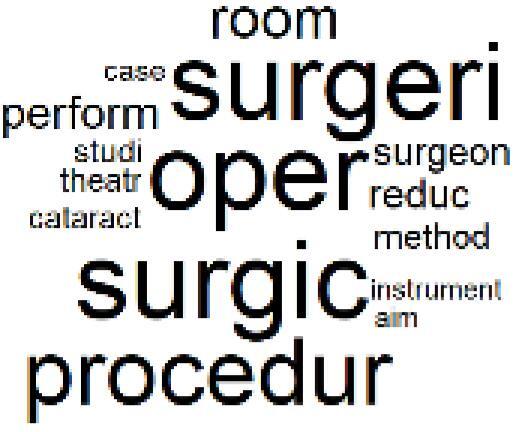	4.0%	39. Community-Based Healthcare Actions	**Figure:** 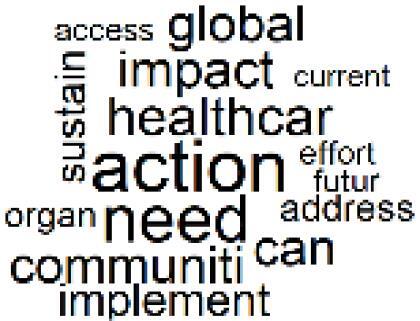	1.2%
20. Drugs and Pharmaceutical	**Figure:** 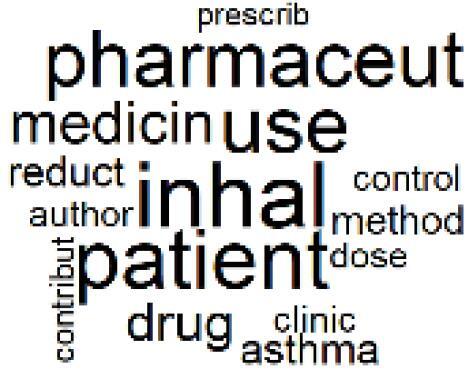	2.8%	40. Hospital Studies and Strategic Planning	**Figure:** 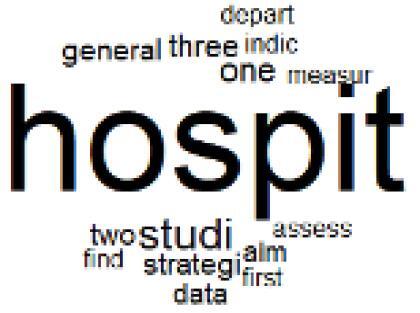	0.5%

Abbreviations: GHG, greenhouse gas; NHS, National Healthcare Service; CE, circular economy.

 The ATM topic modeling grouped papers related to waste management and manufacturing, reuse, and recycling of medical devices into Topics 1, 2, and 3, while studies on green procurement and healthcare supply chains were primarily clustered in Topic 4. Papers addressing environmental impact reduction associated with transportation and telemedicine were categorized into Topics 5 and 6, whereas studies on energy efficiency and renewable energy were clustered in Topics 7 and 8. Sustainable building design papers were grouped in Topic 9, food systems and water use studies in Topics 10 and 11, and healthcare team sustainability awareness was addressed in Topic 12. These scope areas are discussed in Soares et al study.

###  Impact Assessment of Alternative Treatment Pathways and Green Practices

 The ATM topics of 13-18 clustered alternative treatment pathways and green practice papers^39-44^ and the GHG emission accounting,^45-49^ life cycle assessment,^50-52^ and carbon footprint quantification^53-55^ methods were used by the authors. Topic 19 is also about the sustainability of operating room and includes papers related to environmentally sustainable surgical operations.^56-60^ Many instances of pre-2022 related papers^15-28^ were discussed in the previous section.

###  Pharmaceutical Manufacturing, Usage, and Waste 

 Based on Dehipawala et al^1^ study, the production, distribution, and use of pharmaceuticals accounts for 18% of the healthcare sector’s GHG emissions. Such a large contribution justifies the implementation of hospital programs such as drug reuse and take-back initiatives or the purchase of biodegradable drug delivery systems that reduce waste and incineration needs.^61^ The papers in topics 20, 21, and 22 are clustered around the issue of pharmaceutical manufacturing, usage, and waste in the healthcare sector,^62-69^ which was overlooked in the Soares et al paper despite many highly cited papers related to pharmaceutical GHG emissions.^61,70,71^

###  Leveraging Emerging Innovative Technologies

 The clustered papers in Topics 23 and 24 indicate increasing attention in recent years to the use of emerging technologies for implementing CE practices in hospitals.^72-75^ For example, artificial intelligence (AI) has been identified as a tool for optimizing resource allocation to reduce waste and energy consumption.^76,77^ Blockchain technology can enhance transparency within pharmaceutical supply chains and building energy management.^78,79^ Digital twins can support the simulation of processes across economic, environmental, and social dimensions.^80^ In addition, smart sensor technologies enable real-time monitoring of energy use, air quality, and equipment performance, thereby supporting predictive maintenance and waste minimization strategies.^81,82^ Majority of these studies have been published after 2022 and represent an evolving scope area with significant potential to strengthen the integration of CE practices within the healthcare sector.

 After incorporating the three additional scope areas described above, no distinct major category can be identified for Topics 25 through 40, as the papers clustered within these topics generally address the environmental impacts of COVID-19, sustainability–cost trade-offs, health impacts of climate change, the roles of healthcare providers and hospital administrators, and broader research trends related to the environmental impacts of healthcare.

## Conclusion

 In this commentary paper, in addition to expanding the findings of Soares et al by identifying three additional healthcare CE scope areas, the application of machine learning and text mining approaches was examined as a complement to scoping reviews in knowledge domains characterized by large bodies of published literature.

 This study demonstrates the effectiveness of a complementary approach to the standard PRISMA methodology for conducting scoping reviews. The proposed ATM approach can be applied in two ways: either at the beginning of the “Screening” phase to establish an initial knowledge map prior to abstract screening for relevance, or after the “Inclusion” phase to validate the synthesized and identified scope areas. This paper examined the latter approach and expanded the scope areas identified through a standard PRISMA-based study. One potential direction for future research is to replicate this method across other published PRISMA scoping reviews to further demonstrate its effectiveness.

## Disclosure of artificial intelligence (AI) use

 Not applicable.

## Ethical issues

 Not applicable.

## Conflicts of interest

 Author declares that he has no conflicts of interest.
